# Calcifying odontogenic cyst: A 26-year retrospective clinicopathological analysis and immunohistochemical study

**DOI:** 10.4317/jced.54528

**Published:** 2018-06-01

**Authors:** José-Alcides Arruda, Leni-Verônica Silva, Leorik Silva, João-Luiz Monteiro, Pamella Álvares, Marcia Silveira, Ana-Paula Sobral

**Affiliations:** 1 DDS, MSc Student, School of Dentistry, Department of Oral Surgery and Pathology, Universidade Federal de Minas Gerais, Belo Horizonte, MG, Brazil; 2 DDS, MSc, PhD Student, Postgraduate Program in Oral Pathology, Universidade Federal do Rio Grande do Norte, Natal, RN, Brazil; 3 DDS, MSc Student, Departmant of Oral and Maxillofacial Surgery, School of Dentistry, Universidade de Pernambuco, Camaragibe, PE, Brazil; 4 DDS, MSc, PhD Student, Postgraduate Program in Dentistry, School of Dentistry, Universidade de Pernambuco, Camaragibe, PE, Brazil; 5 DDS, PhD, Professor, Postgraduate Program in Dentistry, School of Dentistry, Universidade de Pernambuco, Camaragibe, PE, Brazil

## Abstract

**Background:**

To identify the prevalence and clinicopathological profile of calcifying odontogenic cysts (COC) stored at an oral pathology service, and to analyze the immunoexpression of cyclooxygenase 2 (COX-2) and cyclin D1 (CD1) in these cysts.

**Material and Methods:**

After a retrospective analysis (1990-2016) carried out to identify cases of COC, a sample of 12 cases was selected for immunohistochemical analysis of COX-2 and CD1 by the immunoperoxidase technique. Protein expression was evaluated semiquantitatively by attributing a score of 0 to 3 (0 = no staining; 1 = 1-25%; 2 = 26-50%, and 3 = >51% immunopositive cells).

**Results:**

Twenty cases of COC were diagnosed over the study period. These cysts were more common in the posterior mandible and in men (male-to-female ratio of 1.2:1), with a mean age of 29.9 years. Among the 12 cases analyzed, immunoexpression of COX-2 was observed only in the inflammatory infiltrate in 50% of the cysts (n = 6). Protein CD1 was detected (score 1) in 66.6% of cases (n = 8), and COX-2 was negative in 50% (n = 6).

**Conclusions:**

The prevalence of COC among all odontogenic cysts was 3.5%, representing an uncommon lesion. Immunohistochemical analysis suggested that COX-2 does not participate in lesion progression. The cell proliferation index of COC was low, as demonstrated by the expression of CD1, suggesting a proliferative profile compatible with more indolent lesions.

** Key words:**Odontogenic cysts, odontogenic tumors, epidemiology, immunohistochemistry, cell proliferation.

## Introduction

Calcifying odontogenic cysts (COC) were described for the first time by Rywkind in 1932, but in 1962 Gorlin (1) defined them as an entity pathologically distinct from calcifying odontogenic tumors and characterized them as non-neoplastic cystic lesions. However, in 1981, Praetorius *et al.* (2) proposed a new classification and revised the biological potential of this lesion. In 2005, the World Health Organization (WHO) defined COC as a benign cystic tumor arising from odontogenic epithelium with ectomesenchyme, which can be associated or not with the formation of hard tissue. COC was therefore renamed calcifying cystic odontogenic tumor (3). In 2017, the WHO reclassified this lesion as COC and included it in the group of odontogenic and non-odontogenic developmental cysts (4).

Calcifying odontogenic cysts are uncommon lesions of variable clinical behavior that account for approximately 0.3% of all lesions diagnosed in oral pathology laboratories and for 1 to 7% of odontogenic cysts and tumors (5-7). Thus, knowledge of the biological behavior of lesions affecting the oral cavity, including these cysts, is essential for an adequate therapeutic approach and to establish the prognosis for each case.

The evaluation of cell proliferation can be used as a potential indicator of behavior, treatment response, and recurrence (8). Furthermore, the study of the cell cycle in odontogenic cysts and tumors is important since this is an organized and complex process (7,9). Within this context, cyclin D1 (CD1) is one of the proteins involved in the transition from the G1 to the S phase of the cell cycle in both normal and neoplastic cells. Overexpression of CD1 alters the cell cycle, causing uncontrolled proliferation and transformation to a neoplastic phenotype (10).

In addition to the study of cell proliferation, the analysis of protein expression of cyclooxygenases (COX) in COC can provide information and help establish treatment strategies since the inflammatory response is recognized as one of the first events in tumorigenesis. In this respect, recent studies have demonstrated the participation of COXs in tumor development. COXs are enzymatic mediators of the inflammatory process and are responsible for the conversion of arachidonic acid to prostaglandins and thromboxane. This enzyme is expressed in a limited number of cells and is induced by growth factors and tumorigenic stimuli. Overexpression of COX-2 is related to angiogenesis and cell proliferation (11-13).

To better understand the interaction between cells and biological markers, the objective of this study was to identify the prevalence and clinicopathological profile of COC, and to analyze the immunohistochemical expression of COX-2 and CD1.

## Material and Methods

-Study design, ethical approval and sample

The study was approved by the local Research Ethics Committee (Approval No. 43364215.0.0000.5207). A retrospective study was conducted to identify cases of COC registered at a public oral pathology service in northeastern Brazil between 1990 and 2016. The patient’s identity remained anonymous according to the Declaration of Helsinki.

The sample originated from incisional (n = 9) and excisional (n = 11) biopsies. The clinical data and demographic characteristics were: anatomical site, age at diagnosis, gender, symptomatology, lesion size (determined according to the largest diameter), and radiological aspects were collected from the patients’ medical records.

-Inclusion and exclusion criteria

All included COC cases were classified according the latest edition of the WHO classification (4) as follows: cyst wall: lined with thin ameloblastomatous epithelium; presence of ghost cells: calcified or not; epithelium in adjacent connective tissue: proliferative or not; dysplastic dentin: present or absent. The cases were analyzed by two independent oral and maxillofacial pathologists with more than 20 years of experience. Records without accurate information regarding the histopathological diagnosis were excluded. For immunohistochemical analysis, cases were excluded if there was insufficient material for analysis.

-Immunohistochemistry

For immunohistochemistry, 3-µm thick tissue sections were mounted on organosilane-coated slides. The sections were deparaffinized, rehydrated and immersed in 3% hydrogen peroxide. For antigen retrieval, the sections were heated in 10 mM sodium citrate buffer, pH 6.0, in an electrical pressure cooker (approximately 103 kPa, 120º C, 3 min). The primary antibodies against COX-2 (clone SP21; Spring, Pleasanton, CA, USA, diluted in BSA 1:200) and CD1 (clone P2D11F11; Novocastra, Rockford, IL, USA, diluted in BSA 1:50), then the sections were incubated for 60 min at 4º C. The reaction was amplified with the streptavidin-biotin complex and diaminobenzidine was used as a chromogen for color development. The sections were counterstained with Mayer’s hematoxylin and cover slipped.

Sections of human breast carcinoma were used as positive controls for the anti-COX-2 antibody, and sections of human fetal lung tissue were used for the anti-CD1 antibody. Negative controls were samples treated as described previously, except that the primary antibody was omitted and replaced with non-immune murine IgG1 (X-0931, DAKO) or 1% BSA-PBS for both antibodies studied.

-Evaluation of immunostaining

Immunostaining was analyzed under a light microscope (Nikon Eclipse-E200, Tokyo, Japan) by two oral and maxillofacial pathologists considering the following parameters: type of immunopositive cells and localization (membrane or cytoplasm and nucleus). The observation of a brown color was defined as positive immunostaining.

The pattern of immunoexpression was classified as present or absent. For semiquantitative analysis of immunostaining, a score was attributed to each case according to the percentage of positive cells: 0 (no immunostaining), 1 (1-25% immunostained cells), 2 (26-50% immunostained cells), and 3 (>51% immunostained cells). According to the manufacturers, the localization pattern of positive staining for each protein was: cytoplasmic for COX-2 and nuclear/cytoplasmic for CD1.

-Data analysis

Descriptive and quantitative data analysis was performed using the Statistical Package for the Social Sciences (SPSS) software, version 20.0 (SPSS Inc., Chicago, IL, USA). Pearson’s chi-square test was applied to determine differences in the expression of the proteins in COC. The level of significance was set at *p*≤0.05.

## Results

A total of 6,250 oral and maxillofacial lesions were diagnosed during the study period; of these, 571 (9.1%) were odontogenic cysts and 286 (4.5%) were odontogenic tumors. COC accounted for 0.32% (n = 20) of all cases.

All 20 cases were intraosseous lesions ( Table 1). Of these, 11 samples represented excisional biopsies, and nine were incisional biopsies. There was a male predilection (55%), with a male-to-female ratio of 1.2:1. Patient age ranged from 9 to 58 years (mean: 29.9 ± SD: 18.1 years). With respect to anatomical location of the cysts, the mandible was the most affected site (55%), especially the posterior region. Eighteen (90%) patients were asymptomatic and two (10%) reported painful symptoms.

**Table 1:**
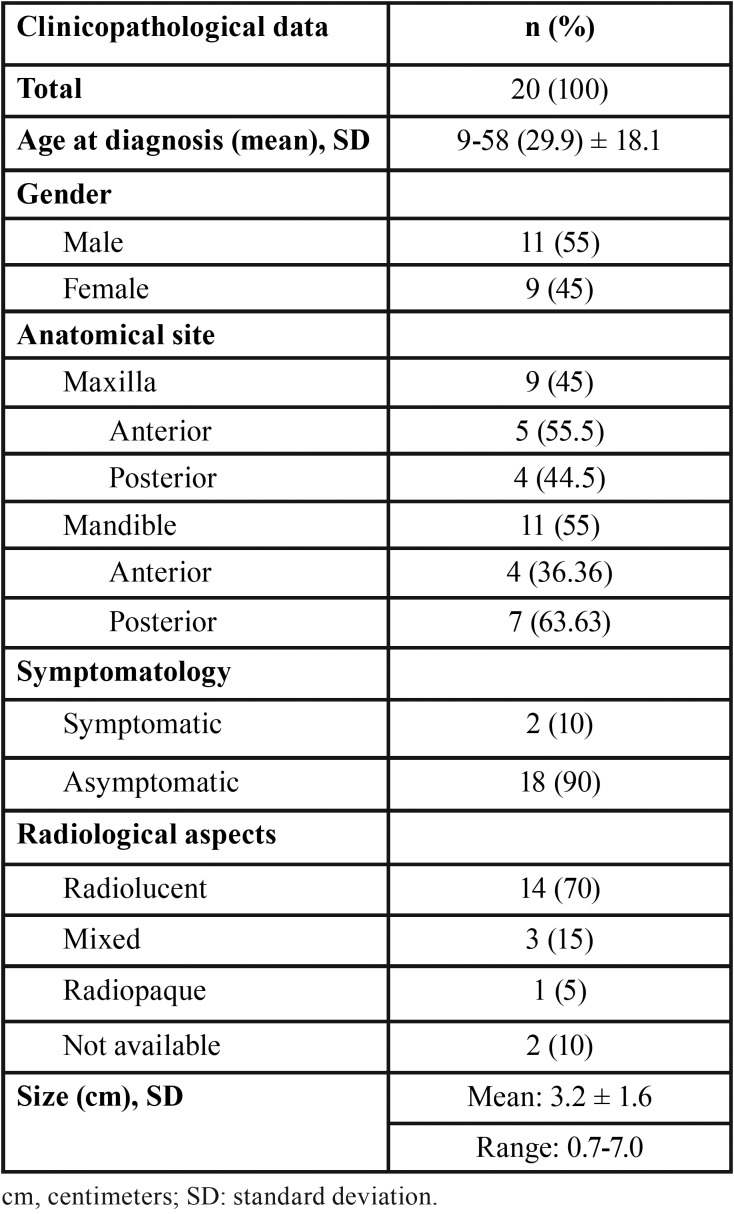
Clinicopathological data of patients with calcifying odontogenic cysts.

Eighteen records (90%) provided a radiographic description of the lesion. Fourteen (70%) had a radiolucent aspect, three (15%) showed mixed aspects and one (5%) displayed a radiopaque aspect. Fifteen cases (75%) showed a unilocular aspect and five (25%) had no information about the locularity of the lesion. Eleven cases (55%) exhibited well-defined radiographic aspects. The mean size of the lesion was approximately 3.2 ± SD: 1.6 cm.

**Figure 1:**
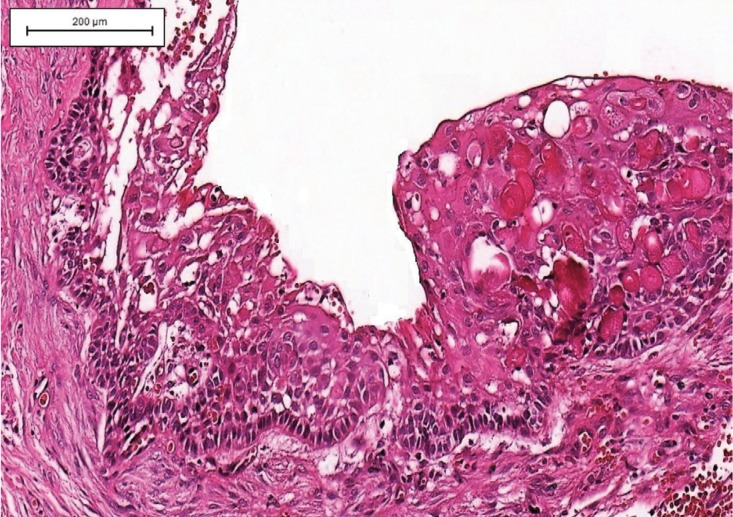
Histopathological features of a calcifying odontogenic cyst. Presence of ameloblastomatous epithelium lining the cystic cavity (H<E; Bars indicate 200µm).

Morphological analysis showed a benign cystic pattern in all 20 cases. The cyst wall was lined with a thin ameloblastomatous epithelium whose basal layer consisted of cubic or columnar cells. Superficially, the cells were loosely arranged, resembling the stellate reticulum of the enamel organ. In addition, variable numbers of slightly eosinophilic epithelial cells without a nucleus, called ghost cells, were observed, being occasionally calcified (Fig. 1). The presence of dysplastic dentin and the proliferation of odontogenic epithelium were noted inside adjacent tissue in half the cases. None of the cases analyzed was associated with odontogenic tumors or other cysts.

Ten (50%) of the 20 cases studied exhibited a predominantly mononuclear inflammatory infiltrate. An inflammatory infiltrate was not evident in the two symptomatic cases; however, these samples were from incisional biopsies. Both COCs were more than 4.0 cm in size. One case was located in the posterior mandible, with duration of symptoms of 7 months and the other was located in the anterior mandible with symptomatology persisting for 12 months.

**Figure 2:**
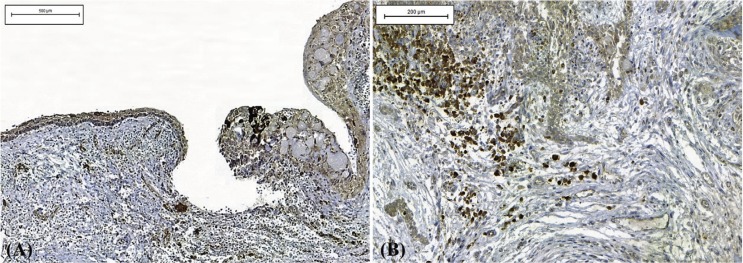
Expression of COX-2 protein in a calcifying odontogenic cyst. (A) Note the immunostaining in the inflammatory cells adjacent to the epithelium lining the cystic cavity with weak immunoexpression (IHC; Bars indicate 500µm). (B) At a higher magnification, strong immunostaining was observed in inflammatory cells (IHC; Bars indicate 200µm).

**Figure 3:**
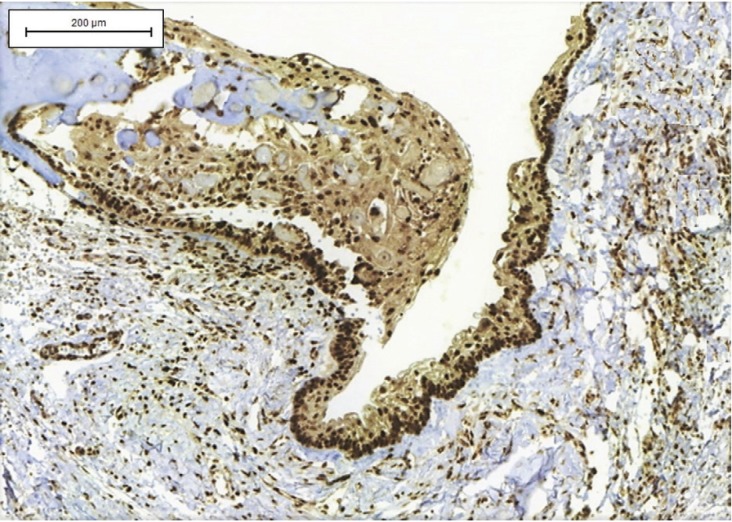
Expression of CD1 protein in a calcifying odontogenic cyst. Observe the nuclear and cytoplasmic immunoexpression in epithelial cells of the basal and parabasal layers of the epithelium lining the cystic cavity (IHC; Bars indicate 200µm).

Immunohistochemical analysis was performed in 12 cases but was not possible in 8 due to insufficient material. In most cases (n = 5, 41.6%), the cysts showed immunoexpression (score 1) of COX-2 in cells of the inflammatory infiltrate adjacent to epithelial proliferation. However, immunostaining for this protein was negative or weak in the epithelial lining of the cystic cavity (Fig. 2). Staining for CD1 was positive in cells of the basal and parabasal layers in 66.6% of cases (n = 8), all of them classified as score 1 (Fig. 3). There were no statistically significant differences in the expression of these proteins in COC ( Table 2).

**Table 2:**
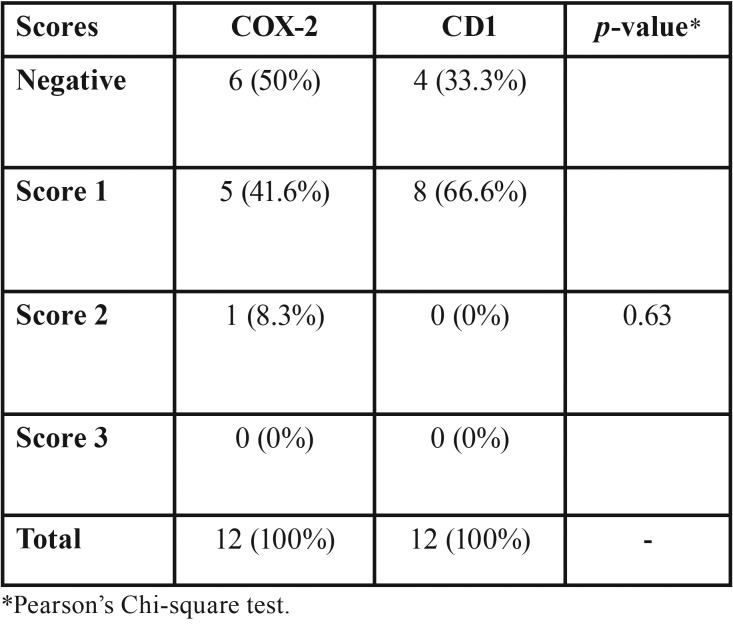
Immunoexpression of COX-2 and CD1 in calcifying odontogenic cysts.

## Discussion

Although no consensus exists regarding the classification and terminology of COC, in 2017, the WHO defined COC as a benign cystic lesion of odontogenic origin that can be classified into two variants: intraosseous and extraosseous (4). However, Praetorius *et al.* (2) suggested to classify this lesion as a cyst or tumor (solid). Three different types can be found in the cystic variant: simple unicystic, unicystic odontoma-producing, and unicystic with ameloblastomatous proliferation.

It is pertinent to note that among the 6,250 diagnoses made at our oral pathology service over a period of 26 years, 20 cases were diagnosed as COC. Our results corroborate the findings of Galana-Alvarez *et al.* (14) who found 0.3% of lesions diagnosed as COC at oral pathology laboratories. Consensus exists that COC is a rare lesion, accounting for 0.37 to 7% of all odontogenic cysts and tumors (6,7,15,16). In this respect, the prevalence of COC was 4.8% among 3,875 cases analyzed in the retrospective study of Mohajerani *et al.* (17).

In the study of Li and Yu (18), 69% of the 21 cases diagnosed as COC occurred in the maxilla and the anterior region was the most affected site (62.5%). In contrast, of the 26 cases of COC identified by Luo and Li (6) among 1,309 odontogenic lesions, 17 involved the maxilla and nine the mandible. In the present study, a higher frequency of COC was observed in the posterior mandible (55%). Information about the anatomical location of the lesion is of fundamental importance for the differential diagnosis since other odontogenic cysts and tumors, and even non-odontogenic cysts and bone diseases such as focal cemento-osseous dysplasia and osteosarcoma, can be found at this site (7).

Geographic variation in the incidence and age distribution of odontogenic cysts and tumors is an important factor that has been discussed in several studies. Since these cysts are often asymptomatic, patients are diagnosed late. According to Buchner *et al.* (5), in developing countries, the number of odontogenic cysts and tumors without a histopathological exam may cause the incidence of these entities to be underestimated. In the study of Avelar *et al.* (19), patients diagnosed with COC were in their second and third decade of life. This finding might be due to the geographic location and different ethnic groups on which the study was conducted. According to Luo and Li (6), COC occurs in a broad age range (3-78 years). In the present study, the mean age of the patients was 29.9 years, a finding that agrees with the epidemiological studies cited above.

da Silva *et al.* (20) found a female predilection of COC in the northeastern Brazilian population, while Fregnani *et al.* (21) suggested no predilection. However, a discrete correlation with female gender is observed when COC involves the maxillary region. In contrast, a male predilection (55%) was observed in the present study. In addition, 18 (90%) of the 20 cases were asymptomatic, in agreement with the findings of Li and Yu (18) and da Silva *et al.* (7).

With respect to histological features, the 2017 WHO classification of odontogenic cysts describes the morphological pattern of the two variants, extra- and intraosseous, to be the same. This pattern includes a cyst wall lined with ameloblastomatous epithelium and the formation of ghost cells that can calcify, as well as epithelial proliferation in adjacent connective tissue and the presence of dysplastic dentin (4). Furthermore, some authors already agree with this current classification and not with the 2005 classification that COC is indeed a neoplasm. Praetorius *et al.* (2) and Iida *et al.* (22) suggested that the cystic (non-neoplastic) forms can be either intra- or extraosseous, in which a well-defined cystic lesion is found and consists of a fibrous capsule and odontogenic epithelial lining of 4 to 10 cells.

Within this context, analysis of the expression of proteins in odontogenic cysts and tumors such as COC is important to understand the molecular mechanism associated with the behavior of these lesions. The expression of COX-2 is induced by different stimuli such as inflammatory signs, cytokines, growth factors, hormones, and tumor promoters. In inflammatory conditions, this protein is able to metabolize prostaglandins (23). In a review of the expression of COX-2 in head and neck tumors, Mendes *et al.* (24) found that the levels of this protein increase in different tumors with aggressive behavior, but that the underlying mechanism is still unknown.

In the present study, positive immunostaining (score 1) for COX-2 protein was observed in 41.6% of cases (n = 5) in which an inflammatory infiltrate was present adjacent to the cystic proliferation, while immunostaining was negative or weak in the cystic epithelium. We believe that a weak immunostaining in the epithelium in a few cases may be due to induction from inflammatory cells that are secreting COX-2. The presence of an inflammatory infiltrate in developmental odontogenic cysts can be attributed to local trauma, aspiration puncture or a previous biopsy. These stimuli are interpreted as aggression by the immune system.

Seyedmajidi *et al.* (25) demonstrated expression of COX-2 in epithelial cells of odontogenic keratocysts and ameloblastomas. When compared to ameloblastomas and odontogenic keratocysts, the absence of COX-2 immunostaining in a large extent of the epithelium of COC suggests a non-neoplastic phenotype of this lesion, as evident in odontogenic tumors. Data on the expression of COX-2 in COC are still sparse. To our knowledge, this is the first study demonstrating the expression of this protein in COC.

Few immunohistochemical studies in the literature have investigated the expression of cyclins in odontogenic cysts and tumors. In the present study, positive staining for CD1 was observed in 66.6% of COC cases (n = 8), all of them classified as score 1, characterizing low proliferative activity (26). In contrast, de Vincent *et al.* (27) reported variability in the expression of CD1 in tumors and cystic lesions of odontogenic origin. The authors observed diffuse nuclear expression in basal and parabasal cells of ameloblastomas and odontogenic keratocysts and low expression in dentigerous cysts. According to our results, the low expression of CD1 in COC may indicate a behavior of these cysts similar to that of a cystic lesion as proposed by the current classification, and not of a neoplasm, considering that cystic lesions are generally less aggressive and have a lower proliferative potential.

Fregnani *et al.* (21) found the Ki-67 immunoexpression indices to be correlated with epithelial proliferation. The authors detected higher proliferative activity in the epithelium of COC when these cysts occurred in association with odontomas and peripheral lesions and concluded that immunohistochemical analysis may be useful to diagnose the variants of COC. However, in the present study, COC was not associated with odontogenic tumors.

Since this was a retrospective study analyzing biopsy records, we do not have data regarding follow-up and recurrence of lesions, a fact that represents a limitation of this study. Therefore, single or multicenter prospective studies of COC lesions are feasible and should be encouraged.

## Conclusions

Calcifying odontogenic cysts are uncommon lesions, with a low prevalence among all odontogenic cysts. Immunohistochemical analysis suggested that COX-2 does not participate in lesion progression and expression of CD1 suggested a proliferative profile compatible with more indolent lesions. Finally, we suggest expanding the panel of markers for further elucidation of the biological behavior of COC.
